# A Nucleosomal Region Important for Ensuring Proper Interactions Between the Transcription Elongation Factor Spt16 and Transcribed Genes in *Saccharomyces cerevisiae*

**DOI:** 10.1534/g3.113.005926

**Published:** 2013-06-01

**Authors:** Hoai-Trang T. Nguyen, William Wharton, Jennifer A. Harper, James R. Dornhoffer, Andrea A. Duina

**Affiliations:** Biology Department, Hendrix College, Conway, Arkansas 72032

**Keywords:** histones, nucleosomes, Spt16, yFACT, transcription elongation

## Abstract

The highly conserved FACT (FAcilitates Chromatin Transactions) histone chaperone assists in the transcription elongation process first by facilitating the removal of histones in front of transcribing RNA polymerase II (Pol II) and then by contributing to nucleosome reassembly in the wake of Pol II passage. Whereas it is well established that FACT localizes across actively transcribed genes, the mechanisms that regulate FACT recruitment to and disengagement from chromatin during transcription still remain to be elucidated. Using the *Saccharomyces cerevisiae* model system, we previously showed that a histone H3 mutant—H3-L61W—greatly perturbs interactions between the yeast FACT (yFACT) complex and chromatin during transcription, resulting in a pronounced shift in yFACT occupancy toward the 3′ ends of transcribed genes. In the present study we report that two histone H4 mutants—H4-R36A and H4-K31E—alter the association pattern of the yFACT subunit Spt16 across transcribed genes in a fashion similar to that seen for H3-L61W. Interestingly, H4-R36, H4-K31, and H3-L61 are in close proximity to each other on the side of the nucleosome. We also provide evidence that the H4-R36A and H3-L61W mutants impair proper Spt16−chromatin interactions by perturbing a common process. Collectively, our results suggest that a nucleosomal region encompassing the H4-R36, H4-K31, and H3-L61 residues plays an important role in ensuring proper association of yFACT across transcribed genes.

Eukaryotic DNA is compacted within the small confines of a cell nucleus through its association with several proteins to form a structure referred to as chromatin. During the initial stages in the formation of chromatin, 147 base pairs of DNA are wrapped around a histone octamer—a protein complex composed of pairs of the four core histone proteins H2A, H2B, H3, and H4—to form a particle known as the nucleosome (Luger *et al.* 1997). Studies from many groups in the last few decades have demonstrated that nucleosomes are not static entities with an exclusive role in DNA compaction but rather have additional central roles in the execution and regulation of most of the processes that occur on DNA. One of the processes for which roles for nucleosomes have been extensively characterized is transcription. During the transcription process, nucleosomes can be manipulated in a variety of ways—for example, through covalent modifications of histone proteins, changes in positioning across DNA regions, or replacement of histone core proteins with histone variants—to ensure proper timing and levels of gene expression (Campos and Reinberg 2009; Rando and Winston 2012).

A prominent focus of recent research in the gene transcription field has been on the understanding of the dynamic functional and physical relationships that exist between nucleosomes and those proteins that assist RNA polymerase II (Pol II) during the elongation phase of transcription. A major task for a subset of these proteins is to first ensure that nucleosomes are partially or completely dismantled in front of transcribing Pol II to facilitate its progression across transcribed units and then to subsequently reassemble nucleosomes after Pol II passage. The proteins involved in these processes carry out their functions through an interplay of a variety of biochemical activities that include nucleosome remodeling, changes in histone posttranslational modifications, and histone chaperoning (Selth *et al.* 2010; Petesch and Lis 2012). The histone chaperone activities that occur during transcription elongation have been attributed to several distinct factors, with the Spt6 and FACT (FAcilitates Chromatin Transactions) histone chaperones being among the better characterized ones. Although both of these factors promote nucleosome reassembly during transcription elongation, the FACT complex also has a well-established role in promoting the disassembly of nucleosomes during elongation to facilitate Pol II access to the underlying DNA (Avvakumov *et al.* 2011; Duina 2011; Winkler and Luger 2011; Formosa 2012).

In the budding yeast *Saccharomyces cerevisiae*, the FACT complex (yFACT) is composed of two protein subunits, Spt16 and Pob3, which, with the assistance of the architectural protein Nhp6, can interact directly with nucleosomes and alter their overall structure (Brewster *et al.* 1998, 2001; Formosa *et al.* 2001; Ruone *et al.* 2003). According to a current model, the physical interaction between yFACT and a nucleosome gives rise to a reorganized nucleosome containing all eight histone proteins, Nhp6, and the yFACT subunits that is more prone to losing H2A-H2B dimers and thus more easily disassembled by a transcribing Pol II as it travels across a gene (Formosa 2008; Xin *et al.* 2009). The ability of yFACT to tether the histone subunits within the context of the reorganized nucleosome is likely to be relevant to the mechanism by which yFACT is also able to reassemble nucleosomes after Pol II passage, a property of FACT that has been supported by several genetic and biochemical studies (Orphanides *et al.* 1999; Formosa *et al.* 2002; Belotserkovskaya *et al.* 2003; Kaplan *et al.* 2003; Schwabish and Struhl 2004; Stuwe *et al.* 2008; VanDemark *et al.* 2008; Jamai *et al.* 2009; Winkler *et al.* 2011).

The mechanisms that regulate the physical interactions that occur between the FACT complex and transcribed genes remain to be fully elucidated. Studies from a variety of model systems have implicated several factors, including the Paf1 complex, the chromatin remodeler Chd1, the histone modifying complex NuA3, and the *Drosophila* HP1 protein as being at least in some cases responsible for the recruitment of FACT to sites of active transcription (Duina 2011). A more recent study has provided evidence for a role of SETD2-mediated trimethylation of histone H3 at lysine 36 (H3-K36me3) in FACT recruitment in HeLa cells (Carvalho *et al.* 2013). FACT is then thought to travel across genes in conjunction with Pol II (Mason and Struhl 2003) and eventually dissociate from chromatin at the end of the transcription process. The mechanisms that control the disengagement of FACT from transcribed genes are yet to be defined, but it appears that these are to a certain extent gene-specific because yFACT has been shown to disengage from certain genes at sites downstream from the polyadenylation (pA) sites and at other genes at sites upstream of the pA sites (Kim *et al.* 2004; Mayer *et al.* 2010).

A more complete understanding of the processes that govern yFACT−chromatin interactions during transcription could be gained by identifying and characterizing any features of the nucleosomes themselves that have roles in controlling the physical interactions between yFACT and transcribed genes. In previous studies, we showed that a specific amino acid substitution within the globular domain of histone H3—H3-L61W—causes a dramatic shift in distribution of Spt16 and Pob3 (yFACT) toward the 3′ ends of several highly transcribed genes as a consequence of lower levels of yFACT abundance at the 5′ ends of these genes and in abnormally elevated levels of yFACT occupancy at the 3′ ends of the same genes (Duina *et al.* 2007; Lloyd *et al.* 2009). These results suggested that the H3-L61W mutant impairs efficient recruitment of yFACT to genes and also cripples the disengagement of yFACT from chromatin at the end of the transcription process. In the present work, we show that two additional histone mutants, in this case both in histone H4 (H4-R36A and H4-K31E), impart similar defects on Spt16−chromatin interactions as those seen in the context of the H3-L61W mutant. H4-R36, H4-K31, and H3-L61 are located in close proximity to each other on the side of the nucleosome, suggesting that these three residues may define a nucleosomal region with a role in directing proper Spt16−chromatin interactions across transcribed genes.

## Materials and Methods

### Yeast strains, genetic methods, and growth media

All *S. cerevisiae* strains listed in Table 1 are *GAL2**^+^* derivatives of the S288C background (Winston *et al.* 1995). Strains yADP67-yADP74 were generated by transforming strain IPY1008 (*MAT**a**his3-205::HIS3-GFP-LacI leu2Δ1::lacO-LEU2ura3-52 trp1Δ63 lys2Δ202 (hht1-hhf1)Δ::KanMX (hht2-hhf2)Δ::clonNAT* <pRS317 (H3-WT/H4-WT)>, a generous gift from Inés Pinto) with pRS414-derived plasmids containing cassettes harboring synthetic histones H3 and H4 genes (*HHTS* and *HHFS*) expressing either wild-type or mutant histone proteins generated by the laboratory of Jef Boeke (Dai *et al.* 2008) followed by loss of the pRS317 plasmid. Note that plasmids pAAD311 and pAAD312 (present in strains yADP71 and yADP67) harbor the H4 base construct and the H3 base construct described by Dai *et al.* (2008), respectively, and both encode wild-type histone H3 and H4 proteins (Dai *et al.* 2008).

**Table 1:** *Saccharomyces cerevisiae* strains

Strain	Genotype
yADP67	*MAT***a** *his3-205*::*HIS3-GFP-LacI leu2Δ1*::*lacO-LEU2 ura3-52 trp1Δ63 lys2Δ202 (hht1-hhf1)Δ*::*KanMX (hht2-hhf2)Δ*::*clonNAT <pAAD312* (H3-WT/H4-WT)*>*
yADP68	*MAT***a** *his3-205*::*HIS3-GFP-LacI leu2Δ1*::*lacO-LEU2 ura3-52 trp1Δ63 lys2Δ202 (hht1-hhf1)Δ*::*KanMX (hht2-hhf2)Δ*::*clonNAT <pAAD303* (H3-K18Q)*>*
yADP69	*MAT***a** *his3-205*::*HIS3-GFP-LacI leu2Δ1*::*lacO-LEU2 ura3-52 trp1Δ63 lys2Δ202 (hht1-hhf1)Δ*::*KanMX (hht2-hhf2)Δ*::*clonNAT <pAAD306* (H3-R49A)*>*
yADP70	*MAT***a** *his3-205*::*HIS3-GFP-LacI leu2Δ1*::*lacO-LEU2 ura3-52 trp1Δ63 lys2Δ202 (hht1-hhf1)Δ*::*KanMX (hht2-hhf2)Δ*::*clonNAT <pAAD307* (H3-G44A)*>*
yADP71	*MAT***a** *his3-205*::*HIS3-GFP-LacI leu2Δ1*::*lacO-LEU2 ura3-52 trp1Δ63 lys2Δ202 (hht1-hhf1)Δ*::*KanMX (hht2-hhf2)Δ*::*clonNAT <pAAD311* (H3-WT/H4-WT)*>*
yADP72	*MAT***a** *his3-205*::*HIS3-GFP-LacI leu2Δ1*::*lacO-LEU2 ura3-52 trp1Δ63 lys2Δ202 (hht1-hhf1)Δ*::*KanMX (hht2-hhf2)Δ*::*clonNAT <pAAD304* (H4-K44A)*>*
yADP73	*MAT***a** *his3-205*::*HIS3-GFP-LacI leu2Δ1*::*lacO-LEU2 ura3-52 trp1Δ63 lys2Δ202 (hht1-hhf1)Δ*::*KanMX (hht2-hhf2)Δ*::*clonNAT <pAAD305* (H4-R36A)*>*
yADP74	*MAT***a** *his3-205*::*HIS3-GFP-LacI leu2Δ1*::*lacO-LEU2 ura3-52 trp1Δ63 lys2Δ202 (hht1-hhf1)Δ*::*KanMX (hht2-hhf2)Δ*::*clonNAT <pAAD313* (H4-K31E)*>*
yADP75	*MAT***a** *his3Δ200 leu2Δ1 ura3**^a^* *lys2-128δ (hht1-hhf1)Δ*::*LEU2 (hht2-hhf2)Δ*:: *HHTS-HHFS-URA3 KanMX4-GAL1pr-FLO8-HIS3*
yADP76	*MAT***a** *his3Δ200 leu2Δ1 ura3**^a^* *lys2-128δ (hht1-hhf1)Δ*::*LEU2 (hht2-hhf2)Δ*:: *HHTS-hhfs*(H4-R36A)*-URA3 KanMX4-GAL1pr-FLO8-HIS3*
yADP77	*MAT***a** *his3Δ200 leu2Δ1 ura3**^a^* *lys2-128δ (hht1-hhf1)Δ*::*LEU2 (hht2-hhf2)Δ*:: *HHTS-hhfs*(H4-R36A)*-URA3 KanMX4-GAL1pr-FLO8-HIS3 SPT16-857*
yADP78	*MAT***a** *his3Δ200 leu2Δ1 ura3**^a^* *lys2-128δ (hht1-hhf1)Δ*::*LEU2 (hht2-hhf2)Δ*:: *HHTS-hhfs*(H4-R36A)*-URA3 KanMX4-GAL1pr-FLO8-HIS3 SPT16-790*
yADP79	*MAT***a** *his3Δ200 leu2Δ1 ura3**^a^* *lys2-128δ (hht1-hhf1)Δ*::*LEU2 hht2-11*(H3-L61W)*KanMX4-GAL1pr-FLO8-HIS3*
yADP80	*MAT***a** *his3Δ200 leu2Δ1 ura3**^a^* *lys2-128δ (hht1-hhf1)Δ*::*LEU2 hht2-11*(H3-L61W) *KanMX4-GAL1pr-FLO8-HIS3 SPT16-857*
yADP81	*MAT***a** *his3Δ200 leu2Δ1 ura3**^a^* *lys2-128δ (hht1-hhf1)Δ*::*LEU2 hht2-11*(H3-L61W) *KanMX4-GAL1pr-FLO8-HIS3 SPT16-790*
yADP82	*MAT***a** *his3Δ200 leu2Δ1 ura3**^a^* *lys2-128δ (hht1-hhf1)Δ*::*LEU2 (hht2-hhf2)Δ*:: *HHTS-hhfs*(H4-R36K)*-URA3 KanMX4-GAL1pr-FLO8-HIS3*

a The allele at this locus is either *ura3-52* or *ura3Δ0*.

The strategy for generating strains yADP75-yADP78 and yADP82 was as follows: the aforementioned pRS414-derived plasmids were digested with the *Bcl*VI restriction enzyme, resulting in the release of the cassettes (which, in addition to the *HHTS-HHFS* genes also include the *URA3* gene) from the plasmid backbones. The digested material was then transformed into strain yAAD859, whose genotype is *MAT**a**his3Δ200 leu2Δ1 ura3-52 trp1Δ63 lys2-128δ (hht2-hhf2)Δ::HIS3* (for a description of the generation of the *(hht2-hhf2)Δ::HIS3* locus, see Duina and Winston 2004) and Ura^+^ transformants were then selected by plating the transformation reaction on solid medium lacking uracil (SC-URA medium). To screen for successful homologous recombination events resulting in the replacement of the *HIS3* gene with the *HHTS-HHFS-URA3* cassettes (note that the ends of the cassettes are homologs to regions flanking the *(hht2-hhf2)Δ::HIS3* locus), the Ura^+^ transformants were then screened for an His^−^ phenotype. Polymerase chain reactions using primers flanking the *HHT2-HHF2* region provided further support that the desired recombination events in selected Ura^+^ His^−^ candidates had in fact taken place. The resulting strains—with the genotype *MAT**a**his3Δ200 leu2Δ1 ura3-52 trp1Δ63 lys2-128δ (hht2-hhf2)Δ::HHTS-HHFS-URA3*, where the synthetic histone-encoding genes express either wild-type histone H4 or one of the two histone H4 mutants, H4-R36A or H4-R36K—were then crossed with preexisting strains and the resulting diploids were sporulated to ultimately give rise to strains yADP75-yADP78 and yADP82. (For a description of the *(hht1-hhf1)Δ::LEU2, KanMX4-GAL1pr-FLO8-HIS3, SPT16-857*, and *SPT16-790* loci present in the strains, refer to Duina and Winston 2004, Duina *et al.* 2007, and Cheung *et al.* 2008). Strains yADP79-81 harbor the *hht2-11* allele, which encodes the H3-L61W mutant, described elsewhere (Duina and Winston 2004).

Techniques for standard genetic experiments have been described by others (Rose *et al.* 1990). Details for the preparation of the growth media used in the experiments presented in this study have been provided previously (Rose *et al.* 1990). The concentrations of the drugs used in the experiments presented in Figure 3 are indicated in the legend of the same figure.

### Genetic screen to identify additional histone mutants that confer H3-L61W−like phenotypes

The yeast histone H3 and H4 mutant library described in the *Results* section was provided by Jef Boeke’s laboratory as frozen aliquots in a series of 96-well microtiter plates. For these experiments, the yeast strains were allowed to partially thaw at room temperature and a small sample of each strain was transferred to 150-mm × 15-mm Petri dishes containing solid YPD medium using a sterilized 96-pronged metal replica plater. Plates were incubated at 30° for 2−3 d to allow for cell growth and were then replica-plated to plates containing YPD, YPD + 15mM caffeine, YPD + 3% formamide, and YPD + 150 mM hydroxyurea media, and incubated at 30° to score for sensitivity to caffeine, formamide, and hydroxyurea. In addition, a set of YPD replica plates was incubated at 14° to score for cold-sensitivity. Strains that showed significant growth defects in the presence of one or more of the drugs used or at 14° were then analyzed further.

### Visualization of the location of specific histone residues on the structure of the nucleosome

The images presented in Figure 2 showing the location of the H4-R36, H4-K31, and H3-L61 residues on the structure of the yeast nucleosome core particle were generated using the PyMOL Molecular Graphics System, Version 1.5.0.3 Schrödinger, LLC using the structure information reported by the laboratory of Karolin Luger (White *et al.* 2001) and available at the Research Collaboratory for Structural Bioinformatics protein data bank (PDB ID:1id3).

### Chromatin immunoprecipitation (ChIP) experiments

ChIP assays were carried out as described previously (Myers *et al.* 2011). These experiments were conducted using 1 μL of the following antibodies: rabbit polyclonal antibody specific for the Spt16 protein (a generous gift of Tim Formosa), a mouse monoclonal antibody specific to the yeast Pol II subunit Rpb3 (Neoclone, no. W0012), and a rabbit polyclonal antibody specific for bulk histone H3 (ab1791; Abcam). The following primers used for the *PMA1* and NO ORF regions have been previously described (Myers *et al.* 2011): 5′*PMA1*: OAD394 and OAD395; internal *PMA1*: OAD416 and OAD417; 3′*PMA1*: OAD383 and OAD384; NO ORF: OAD377 and OAD378. The following primers have not been previously described: 5′*FBA1*: OAD419 (5′ CGCTGCTTTAGAAGCTGCTAGA) and OAD420 (5′ CCACCGTTAGAGGTTTGCAAA); internal *FBA1*: OAD421 (5′ CAAGGCTTTGCACCCAATCT) and OAD422 (5′ CCGTGACAGTTACCGAAAGCA); 3′*FBA1*: OAD423 (5′ GGTCGGCTCTTTTCTTCTGAAG) and OAD424 (5′ AAATAGTGCATGACAAAAGATGAGCTA); 5′*GAL1*: OAD442 (5′ CCTGAGTTCAATTCTAGCGCAAA) and OAD443 (5′ TCTTAATTATGCTCGGGCACTTT); 3′*GAL1*: OAD444 (5′ CCCTTTGTCCTACTGATTAATTTTGTACT) and OAD445 (5′ TCCTCCTCGCGCTTGTCT); 5′*GAL2*: OAD446 (5′ CCAAGCTGGTGAAGACGTGAT) and OAD447 (5′ TTTTGAGATTGTGCGCTTAAATG); and 3′*GAL2*: OAD448 (5′ CGAGCAAAATTAAAAACGCAAA) and OAD449 (5′ GATAAGTCTGGTGATGTGGTCCTTT). For each quantitative polymerase chain reaction experiment, control reactions were conducted to ensure linearity of the results and to determine the efficiency of the primer set used.

## Results

### A genetic screen identifies two histone H4 mutants that perturb association of Spt16 with transcribed genes

In addition to causing a shift in distribution of yFACT toward the 3′ ends of transcribed genes, the H3-L61W mutant confers additional defects, including cold-sensitivity (Cs^−^) and sensitivity to the drugs caffeine, hydroxyurea, and formamide (Caff^s^, HU^s^, and Form^s^, respectively) (Duina and Winston 2004; Myers *et al.* 2011). In an attempt to identify additional histone mutants that perturb yFACT association with chromatin in a manner similar to that seen in H3-L61W cells, we first screened a yeast library of histone H3 and histone H4 mutants generated and kindly provided to us by the laboratory of Jef Boeke (Dai *et al.* 2008) for H3-L61W−like phenotypes (Cs^−^, Caff^s^, HU^s^, and Form^s^—note that many of the strains screened in this study have also been screened previously for some of these phenotypes by others—Dai *et al.* 2008; Huang *et al.* 2009). This library includes the 486 yeast strains in the S288C background described in the original study (Dai *et al.* 2008) that contain integrated synthetic genes expressing histone proteins with single amino acid substitutions at each residue (in most cases, nonalanine residues were changed to alanine and alanine residues were changed to serine, but some other substitutions were also included in the library), plus nearly 100 more mutants that express histone H3 and H4 proteins with additional types of amino acid substitutions that were generated later by the Boeke laboratory. (Note that these and all the other strains used in this study have been engineered to express histones H3 and H4 each from a single gene; see Table 1.) From this screen, seven histone mutants were identified as conferring at least a subset of H3-L61W phenotypes and were considered further.

Subsequent genetic manipulations suggested that some of the seven histone mutant strains had likely diploidized at some point during their propagation since crosses between them and haploid partners generated cells that produced a large proportion of inviable spores after induction of meiosis, a phenomenon usually seen in meiotic products derived from triploid cells. To circumvent this problem, we transformed plasmids expressing the same histone mutants (also kindly provided by the Boeke laboratory) into a new host strain, eventually resulting in freshly-generated yeast strains each expressing one of the seven histone mutants from a plasmid. One of the histone mutants appeared unable to support detectable growth when expressed from a plasmid in our host strain background and was not further examined. The phenotypes of the remaining six strains were tested and compared to those seen in the original strains from the histone mutant library. As can be seen in Table 2, whereas some of the phenotypes were similar between the library strains and the strains expressing the same mutants from plasmids, some were different. For example, the library strain expressing the H4-K31E mutant showed a strong Caff^s^ phenotype, whereas the same mutant expressed from a plasmid in our host strain showed essentially no Caff^s^ phenotype. These differences may be attributable to (1) elevated expression of the mutant proteins in the plasmid-containing strains caused by the presence of more than one plasmid in these cells, (2) the acquisition of additional spontaneous mutations in the library strains during their propagation, and/or (3) differences in the genetic background between the strain used for the construction of the mutant histone library strain and the host strains we used for the plasmid experiments.

**Table 2:** Phenotypic analysis of specific histone H3 and histone H4 mutants

	Phenotypes of Strains Obtained from the Histone Mutant Library*^a^*					Phenotypes of Strains in Which Histone Mutants Are Expressed from Plasmids*^a^*					
	YPD	YPD	YPD	YPD	YPD	YPD	YPD	YPD	YPD	YPD	
Histone		14°	Caff.	Form.	HU		14°	Caff.	Form.	HU	Phenotypes, Dai *et al.**^b^*
WT	5*^c^*	5	5	5	5	5	5	5	5	5	
H3-K18Q	4	4	3	4	5	5	5	5	5	5	
H3-R49A	5	2	0	4	3	3	2	0	1	2	HU^s^
H3-G44A	5	4	1	4	2	2	1	0	0	1	HU^s^
H4-K44A	5	3	3	4	3	5	4	3	5	5	
H4-R36A	5	0	1	2	1	3	0	2	0	2	Cs^−^, HU^s^
H4-K31E	4	4	1	4	1	5	4	5	5	5	n/d

a Phenotypes were scored based on data obtained from spot tests similar to those shown in Figure 3. Refer to the text for a description of the difference between the two sets of strains indicated here.

b See Dai *et al.* (2008) and Huang *et al.* (2009). The only phenotypes considered here are cold-sensitivity (scored at 16°) and HU-sensitivity conferred by the indicated mutants when integrated into the genome and/or expressed from a plasmid. Dai *et al.* (2008) did not score for caffeine or formamide sensitivity.

c Growth was scored on a scale of 5 to 0, with 5 indicating robust growth similar to that seen in wild-type cells and 0 indicating no detectable growth.

**Figure 1:**
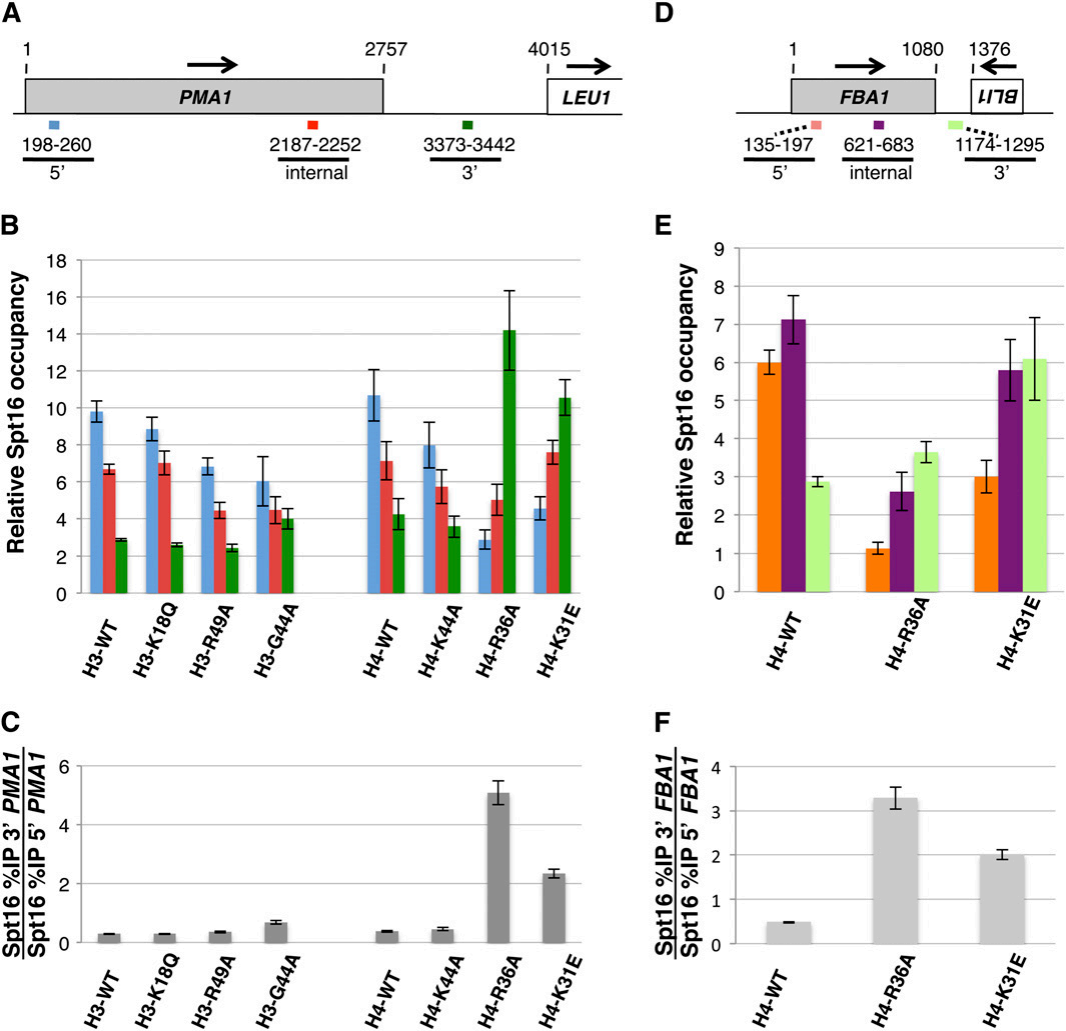
The H4-R36A and H4-K31E mutants cause pronounced perturbations in the distribution of Spt16 across the *PMA1* and *FBA1* genes. (A, D) Schematic representations, roughly drawn to scale, of the *PMA1* and *FBA1* genes and the respective downstream genes. Arrows indicate the direction of transcription for each gene and the numbers correspond to nucleotide positions, with nucleotide 1 corresponding to the first nucleotide of the open reading frame (ORF) of either the *PMA1* gene or the *FBA1* gene, both of which are represented as shaded rectangles in the diagrams. The colored lines below each diagram represent the regions—5′, internal, and 3′—assayed in the ChIP experiments. (B) Results from ChIP experiments in which the occupancy of the Spt16 protein across the *PMA1* gene was assayed in strains expressing the indicated histone genes (the strains used in these experiments are yADP67-yADP74). Relative Spt16 occupancy in each case was determined by dividing the %-immunoprecipitation (%IP) value for the specific region of interest to the %IP value for a nontranscribed region on chromosome V (NO ORF region; see *Material and Methods* for the primers used for this region). For each strain, the data are presented as the mean ± SEM from at least three independent experiments. As a reference, the NO ORF %IP values for each strain were as follows: yADP67: 0.14 ± 0.005, yADP68: 0.16 ± 0.013, yAADP69: 0.27 ± 0.021, yAADP70: 0.24 ± 0.025, yADP71: 0.16 ± 0.029, yADP72: 0.19 ± 0.046, yADP73: 0.24 ± 0.053, yADP74: 0.12 ± 0.018. (C) Data from the experiments described in (B) but represented as the ratio between the %IP value for the 3′ region of *PMA1* and the %IP for the 5′ region of *PMA1*. Representation of the data in this fashion facilitates the assessment of the overall effects imparted by each histone mutant on the distribution of Spt16 across the *PMA1* gene. (E, F) Effects of the H4-R36A and H4-K31E mutants on the levels of Spt16 occupancy across the *FBA1* gene. The data in these panels are presented as described in (B) and (C).

Strains expressing the six histone mutants from plasmids were then subjected to ChIP assays to determine whether any of them cause abnormal distribution of the yFACT component Spt16 across the constitutively transcribed gene *PMA1*. In these experiments we found that of the six mutants, H4-R36A and H4-K31E cause a pronounced perturbation in Spt16 distribution across this gene compared with wild-type cells (Figure 1, A−C). Specifically, the H4-R36A and H4-K31E mutants cause a 3′ shift in Spt16 occupancy at this gene as a result of higher levels of Spt16 over the 3′ end of the gene compared to the 5′ end—a pattern that is the reverse of what normally occurs in histone wild-type cells. Similar perturbations on Spt16-chromatin association by the H4-R36A and H4-K31E mutants were also seen at *FBA1*, another constitutively expressed gene (Figure 1, D−F), showing that the effects of these histone H4 mutants on Spt16−chromatin interactions are not specific for the *PMA1* gene but are likely to be more widespread.

### The H4-R36 and H4-K31 residues are located in close proximity to each other and to the H3-L61 residue on the structure of the nucleosome

**Figure 2:**
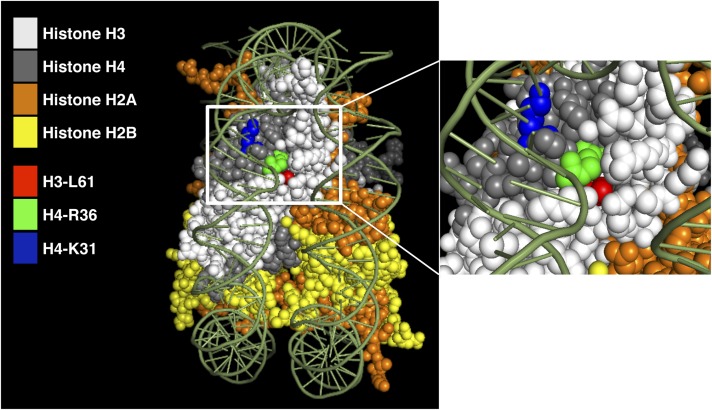
The H4-R36, H4-K31, and H3-L61 residues are located in close proximity to each other on the side of the nucleosome. The left panel shows a side-view of the structure of the yeast nucleosome core particle with the histone proteins and the three histone residues of interest color-coded as indicated and the DNA colored in green. The right panel shows a close-up view of the region boxed by the white rectangle in the left panel that includes the H4-R36, H4-K31, and H3-L61 residues.

The H4-R36A and H4-K31E mutants affect Spt16 distribution across the *PMA1* and *FBA1* genes in a manner similar to that we have previously reported for the H3-L61W mutant (Duina *et al.* 2007). In an attempt to gain insights into how these different mutants cause similar impairments on Spt16−chromatin interactions, we mapped the locations of the three wild-type residues onto the yeast core nucleosome structure (White *et al.* 2001) and compared their positions in relation to each other. As shown in Figure 2, the H4-R36, H4-K31, and H3-L61 residues are located in striking vicinity to each other on the side of the nucleosome, with the H3-L61 residue buried toward the interior of the nucleosome and the H4-R36 and H4-K31 residues exposed on the outer surface. The close proximity of these three residues to each other on the structure of the nucleosome and the similar effects of mutations at these residues on the localization of Spt16 across the *PMA1* and *FBA1* genes suggest that the H4-R36, H4-K31, and H3-L61 residues may define a region of the nucleosome with a role in controlling Spt16-chromatin interactions during transcription.

### Genetic tests indicate that the H4-R36A and H3-L61W mutants affect cell functions through perturbations of common processes

The H4-R36 and H3-L61 residues appear to be particularly important in controlling Spt16 interactions with transcribed genes since specific amino acid substitutions at these location—H4-R36A and H3-L61W—cause the most significant defects in the distribution of Spt16 across transcribed genes (Figures 1 and 4 and Duina *et al.* 2007). Based on these observations, we decided to continue our characterization of the nucleosomal region under investigation by directing our studies on the H4-R36A mutant, with a particular focus on its relationship with the better-characterized H3-L61W mutant.

**Figure 3:**
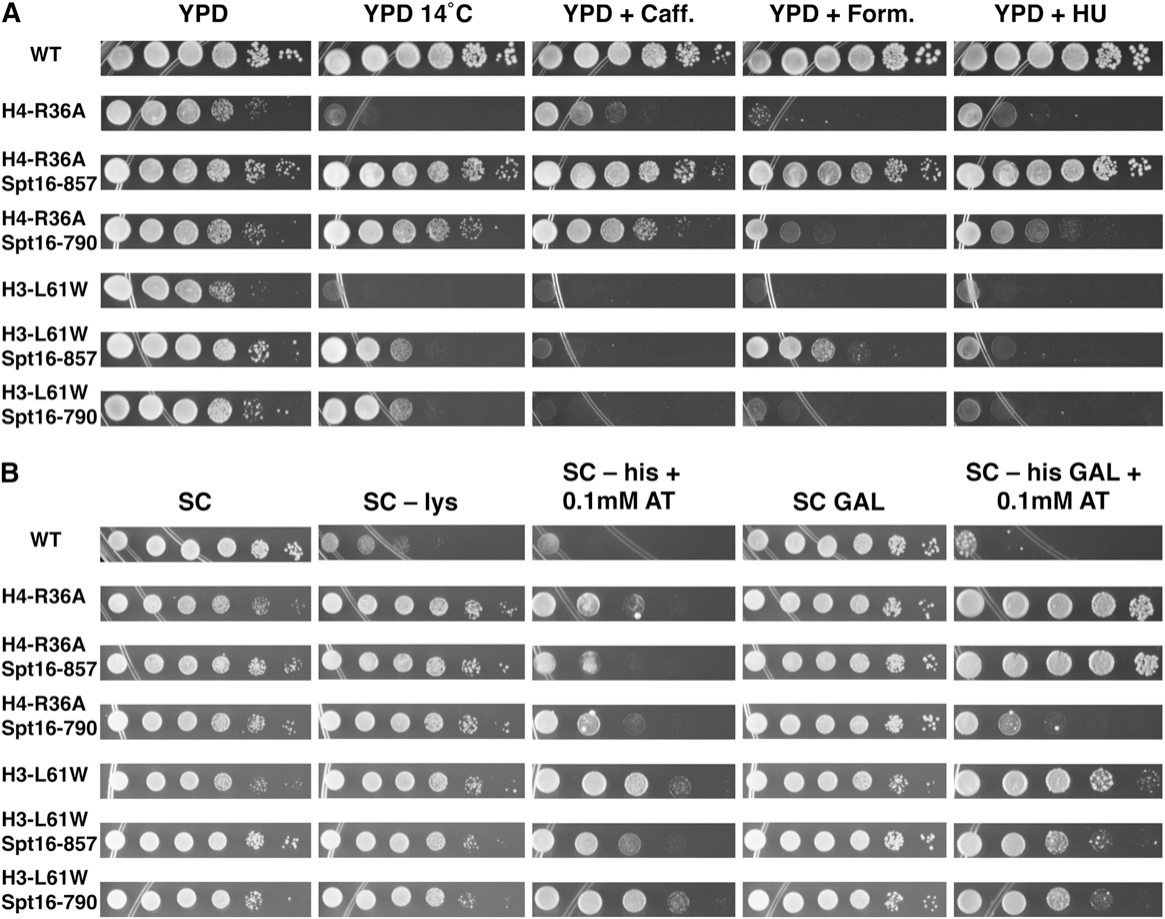
Phenotypic assays indicate that the H4-R36A and H3-L61W mutants affect common cellular processes. (A) Cells expressing wild-type or mutant histones and either wild-type or mutant Spt16 proteins, as indicated, were grown to saturation on YPD medium, harvested, and then spotted on the indicated media in 10-fold dilution series, in which the most concentrated spots (left-most spot in each panel) contained ~2 × 10^6^ cells. The plates were incubated at 30° (except for the plates shown in the second column, which were incubated at 14° as indicated) and photographs were taken after the following approximate times of incubation: YPD, 2 d; YPD 14°, 13 d; YPD + 15mM caffeine (Caff.), 4 d; YPD + 3% formamide (Form.), 4 d; YPD + 150mM hydroxyurea (HU), 4 d. The strains used in these experiments were yAAD75-yAAD81. (B) The same strains used in the experiments described in (A) but spotted on different media, as indicated, such that the most concentrated spots contained ~4 × 10^6^ cells. The plates were incubated at 30° and were photographed after the following approximate times of incubation: SC, 2 d; SC–lysine (lys), 3 d; SC–histidine (his), and with 0.1mM aminotriazole (AT, a competitive inhibitor of the product of the *HIS3* gene), 5 d; SC with galactose (GAL), 3 d; SC–histidine, with galactose and 0.1mM aminotriazole, 5 d. All the media used in these experiments contained glucose as the carbon source, except where otherwise indicated.

Whereas cells expressing H4-R36A display several growth phenotypes in common with those seen in H3-L61W cells (Table 2, Figure 3, and Duina *et al.* 2007 and Myers *et al.* 2011), the mechanistic defects underlying these phenotypes may not necessarily be the same for the two histone mutants. To determine whether the phenotypes conferred by the H4-R36A and H3-L61W mutants are due to perturbations in common or different processes, we performed a series of genetic experiments comparing the phenotypes of H4-R36A and H3-L61W cells in different genetic backgrounds. To facilitate genetic analyses and to eliminate the possibility of artifacts due to changes in gene copy number of histone genes expressed from plasmids, for these and subsequent experiments we generated strains in which the genes expressing the histone mutants are integrated into the genome of a common host strain at their endogenous locations (see *Materials and Methods* and Table 1).

Consistent with the data shown in Table 2, a mutant strain expressing H4-R36A from an integrated gene in our host strain displays several growth phenotypes, including a slow-growth phenotype under permissive conditions and Cs^−^, Caff^s^, Form^s^, and HU^s^ phenotypes (Figure 3A). All of these phenotypes are in common with H3-L61W cells (Figure 3A and Duina *et al.* 2007; Myers *et al.* 2011). As a way to determine whether the H4-R36A mutant confers these phenotypes by perturbing the same cellular processes that are perturbed by the H3-L61W mutant, we asked whether two independent mutations in the Spt16 protein previously shown to suppress H3-L61W-phenotypes (Duina *et al.* 2007) are also able to suppress H4-R36A phenotypes. As shown in Figure 3A, these mutants—Spt16-E857Q and Spt16-E790K (referred to as Spt16-857 and Spt16-790, respectively)—were found to suppress H4-R36A phenotypes in a manner similar to that seen in the context of the H3-L61W mutant. It is worth noting that H4-R36A appears to be generally a “weaker” mutant than the H3-L61W mutant because although the two mutants share similar phenotypes, those displayed by H4-R36A cells are generally milder and are more easily suppressed by the Spt16 mutants. Nevertheless, the fact that H4-R36A and H3-L61W cells can be suppressed in a similar fashion by the same set of mutations is consistent with the notion that the two histone mutants affect cell functions through perturbations of common processes.

### Comparison of the effects of H4-R36A and H3-L61W on phenotypes indicative of transcription and chromatin defects

To more fully characterize the H4-R36A mutant and to further compare it with the H3-L61W mutant, we tested cells expressing each of the histone mutants in the context of wild-type or mutant Spt16 proteins for phenotypes associated with transcription and chromatin defects. In the first set of experiments, we assayed for Spt^−^ phenotypes by testing cells harboring the *lys2-128δ* allele for their ability to grow in the absence of lysine in the growth medium (for a description of Spt^−^ phenotypes, see Winston 1992). As previously reported, both H4-R36A and H3-L61W cells displayed an Spt^-^ phenotype (Duina *et al.* 2007; Dai *et al.* 2008), and neither histone mutant strains were found to be significantly suppressed for this phenotype by either Spt16 mutants (Figure 3B).

In the second set of experiments, we used a reporter construct to indirectly assay for defects in nucleosome reassembly during transcription elongation (Cheung *et al.* 2008). In this reporter system, the *HIS3* gene is fused to a cryptic promoter within the coding region of the *FLO8* gene and the *FLO8* promoter has been replaced with the inducible *GAL1* promoter. With this reporter, improper nucleosome reassembly and the resulting intragenic cryptic transcription initiation can easily be monitored by testing the ability of cells (deleted for their endogenous *HIS3* gene) to grow on media lacking histidine under conditions in which the *GAL1* promoter is either activated or repressed. As shown in Figure 3B, H4-R36A cells had modest growth on medium lacking histidine and containing glucose and much stronger growth on medium lacking histidine and containing galactose, indicating that cryptic initiation occurs in H4-R36A cells and that this defect is much more pronounced in the context of elevated levels of transcription. [It is worth noting that in another study, cryptic initiation events were not detected at the endogenous *FLO8* gene in H4-R36A cells using Northern blotting (Hainer and Martens 2011)—the discrepancy between these results and ours could be due to differences in the level of sensitivity of the assays used in the two studies or might reflect an authentic difference in the effect of the H4-R36A in the context of the *FLO8-HIS3 vs.* that of the endogenous *FLO8* gene]. Consistent with previous results (Myers *et al.* 2011), H3-L61W cells also grew on media lacking histidine in the presence of either glucose or galactose, indicating that this histone mutant also causes cryptic intragenic transcription at *FLO8-HIS3* under both conditions of high transcription levels and of marginal to no transcription (Figure 3B). The patterns of suppression of the cryptic transcription phenotypes by the two Spt16 mutants were found to be generally similar for H4-R36A and H3-L61W cells, with the notable exception of the Spt16-790 mutant, which under conditions of high transcription strongly suppresses the cryptic initiation phenotype of H4-R36A cells but only modestly suppresses the same defect in H3-L61W cells (Figure 3B).

Taken together, the results from these experiments show that H3-L61W and H4-R36A confer similar phenotypes associated with transcription and chromatin defects and that one of these phenotypes (that seen in the context of the *FLO8-HIS3* reporter construct) can be partially suppressed by the same two Spt16 mutants, albeit at varying levels of efficacy.

### Evidence that H4-R36A affects the pattern of association of Spt16 across the *PMA1* and *FBA1* genes by perturbing the same process that is also perturbed by the H3-L61W mutant

**Figure 4:**
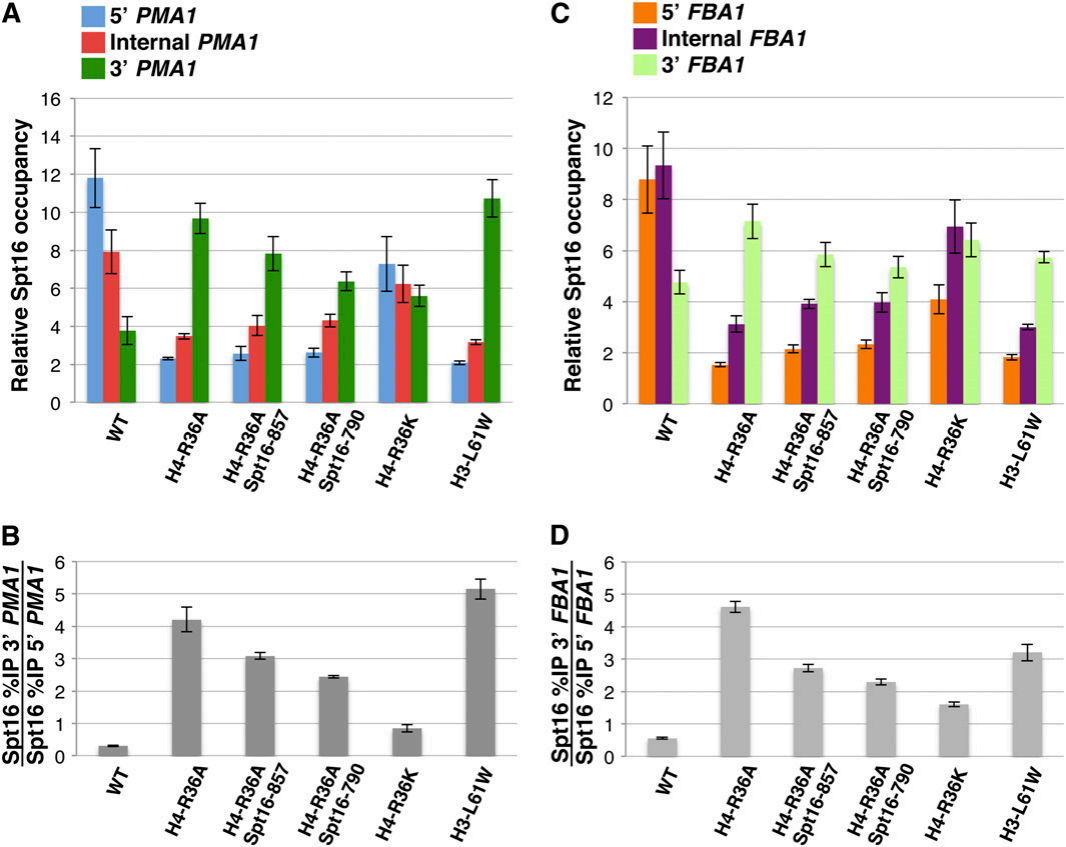
The H4-R36A−dependent defects in Spt16 distribution across the *PMA1* and *FBA1* genes are partially suppressed by two Spt16 mutants and an H4-R36K mutant causes some perturbations in Spt16 interactions with the same genes. (A−D) Results from Spt16 ChIP experiments performed on the indicated strains are presented using the same methodologies described in Figure 1. The three regions (5′, internal, and 3′) assayed for each of the two genes are the same as those shown in Figure 1. The strains used in these experiments were yADP75-79 and yADP82. For each strain, the data are presented as the mean ± SEM from five independent experiments. As a reference, the NO ORF %IP values for each strain were as follows: yADP75: 0.18 ± 0.050, yADP76: 0.25 ± 0.038, yADP77: 0.29 ± 0.049, yADP78: 0.33 ± 0.075, yADP82: 0.22 ± 0.043, yADP79: 0.75 ± 0.257. Note that the mean value for Spt16 binding to the NO ORF region in H3-L61W cells (yADP79) is greater than what is seen in the other strains. Although in these particular experiments the SEM is quite high (making the mean level of Spt16 NO ORF binding in H3-L61W cells not statistically different from the mean level of Spt16 NO ORF binding in wild-type cells), in previous studies we have seen a consistent trend in which Spt16 binding to the NO ORF region in H3-L61W cells appears to be ~2-fold greater than what is normally seen in wild-type cells (Duina *et al.* 2007; Lloyd *et al.* 2009; Myers *et al.* 2011).

We sought to determine whether the phenotypic characteristics shared by H4-R36A and H3-L61W cells discussed previously were reflective of a common mechanism by which these two histone mutants perturb Spt16 association with transcribed genes. To do this, we asked whether the Spt16-790 and Spt16-857 mutants, which had been previously shown to partially suppress the Spt16 distribution defect in H3-L61W cells (Duina *et al.* 2007), could also suppress the defects in Spt16 occupancy across the *PMA1* and *FBA1* genes caused by the H4-R36A mutant. As shown in Figure 4, both Spt16 mutants are in fact able to partially suppress the H4-R36A-dependent shift in Spt16 distribution across both genes. Interestingly, the Spt16-790 has a more pronounced suppressive effect than Spt16-857, a finding that parallels what is seen in the context of the H3-L61W mutant (Duina *et al.* 2007). It is also noteworthy that H4-R36A and H3-L61W affect Spt16 occupancy across both genes to similar degrees. In these experiments we also found that an H4-R36K mutation causes some defect in Spt16 association with chromatin, in particular at the *FBA1* gene, indicating that an aspect of the arginine residue other than its basicity is important for its participation in this process. Collectively, these results indicate that the H4-R36A and H3-L61W mutants affect Spt16 association with transcribed genes to a similar extent and that both likely perturb a common process normally required for proper Spt16−chromatin interactions during transcription.

### The alteration in Spt16 distribution across genes seen in H4-R36A cells appears to be dependent on active transcription

**Figure 5:**
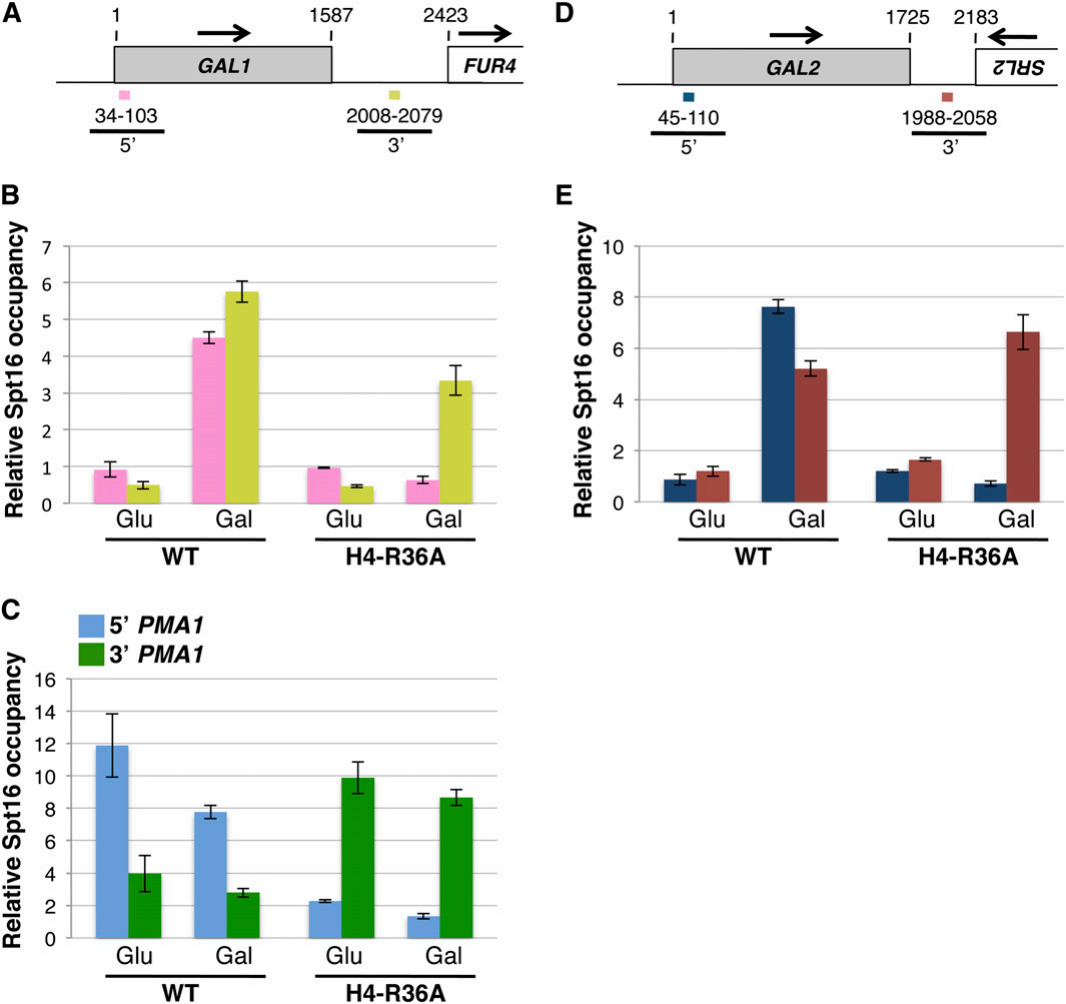
The H4-R36A mutant causes pronounced defects in Spt16 association across the *GAL1* and *GAL2* genes but only under conditions that activate these genes. (A, D) Schematic representations of the *GAL1* and *GAL2* genes using the same numbering and labeling systems as those described in Figure 1. (B, C, E) Cells expressing wild-type histone H4 (WT) or the H4-R36A mutant grown in medium containing either glucose (Glu) or galactose (Gal) were subjected to Spt16 ChIP assays, and levels of Spt16 occupancy at the 5′ and 3′ locations of the *GAL1* and *GAL2* genes indicated in (A) and (D) and at the 5′ and 3′ locations of *PMA1* indicated in Figure 1 were determined. The results are presented using the same methodology as that described in Figure 1. The strains used in these experiments were yADP75 and yADP76. For each strain, the data are presented as the mean ± SEM from three independent experiments. As a reference, the NO ORF %IP values for each strain were as follows: yADP75: 0.23 ± 0.072 (Glu) and 0.58 ± 0.186 (Gal), yADP76: 0.23 ± 0.054 (Glu) and 0.82 ± 0.088 (Gal). Note that some of the data shown in this figure correspond to a subset of the data already presented in Figure 4.

We have previously shown that the H3-L61W mutant markedly affects the distribution pattern of Spt16 across the *GAL1* and *GAL2* genes but only under conditions in which these genes are being actively transcribed (*i.e.*, in the presence of galactose), thus indicating that the effects of H3-L61W on Spt16 interactions with chromatin across genes are dependent on transcription (Duina *et al.* 2007). To determine whether H4-R36A also affects Spt16-gene interactions in a transcription-dependent fashion, we carried out ChIP experiments in wild-type and H4-R36A cells grown in either glucose or galactose medium and determined the level of Spt16 occupancy across the *GAL1* and *GAL2* genes. As shown in Figure 5B, whereas the background pattern of Spt16 occupancy across the *GAL1* gene is indistinguishable in wild-type and H4-R36A cells grown under repressive conditions, Spt16 association pattern across *GAL1* under activating conditions is significantly affected by the H4-R36A mutant, resulting in an overall shift in Spt16 occupancy toward the 3′ end of the *GAL1* gene. A similar but more striking effect is also seen at the *GAL2* gene (Figure 5E). As expected, we detected abnormal Spt16 association across the constitutively expressed gene *PMA1* in H4-R36A cells regardless of the carbon source used in the experiment (Figure 5C). The data from these experiments are consistent with the notion that, similarly to H3-L61W, the H4-R36A mutant causes perturbations in Spt16 association across transcribed genes in a manner that is dependent on active transcription.

### The H4-R36A mutant confers modest defects in the distribution of Pol II and nucleosomes across the *PMA1* and *FBA1* genes

**Figure 6:**
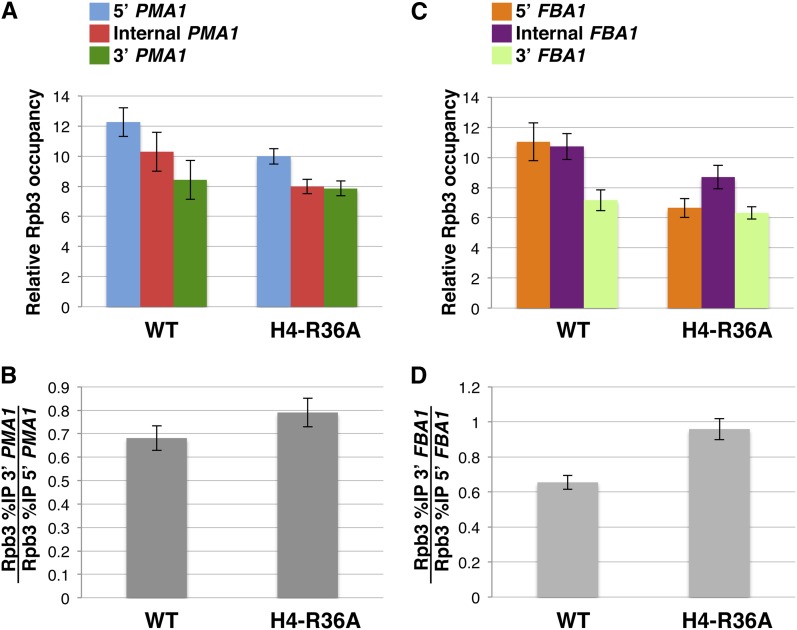
Cells expressing the H4-R36A mutant show minimal perturbations in the association of the Pol II component Rpb3 across the *PMA1* and *FBA1* genes. (A−D) Results from ChIP experiments in which the distribution of Rpb3 across the *PMA1* and *FBA1* genes was assayed in cells expressing either wild-type histone H4 (WT) or the H4-R36A mutant. The strains used in these experiments were yADP75 and yADP76. The results are presented using the same methodology as that described for the experiments shown in Figure 1. For each strain, the data are presented as the mean ± SEM from three independent experiments. As a reference, the NO ORF %IP values for each strain were as follows: yADP75: 0.03 ± 0.007, yADP76 and 0.02 ± 0.005.

**Figure 7:**
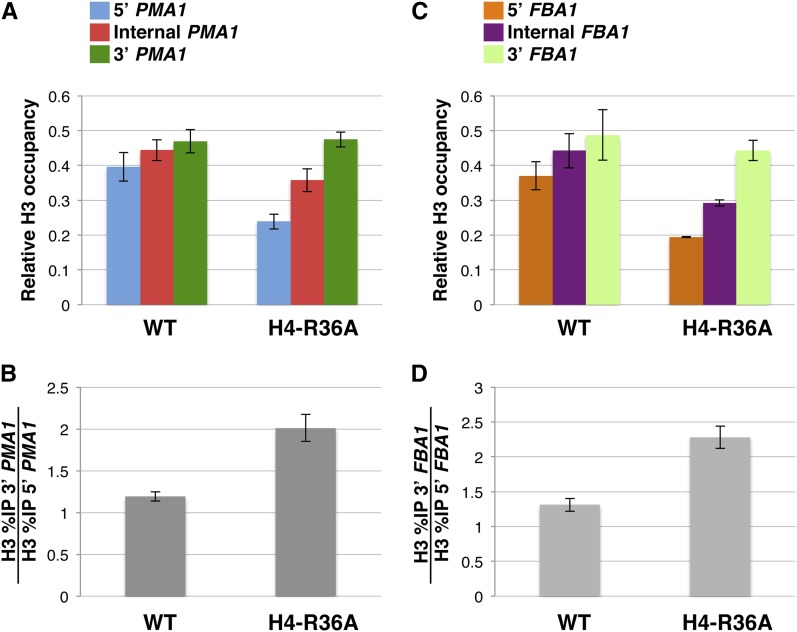
The H4-R36A causes a modest change in bulk nucleosome distribution across the *PMA1* and *FBA1* genes. (A−D) ChIP experiments were carried out on cells expressing either wild-type histone H4 (WT) or the H4-R36A mutant using an antibody that recognizes bulk histone H3. The strains used in these experiments were yADP75 and yADP76. The results from these experiments are presented using the same methodology as that described in the legend to Figure 1. For each strain, the data are presented as the mean ± SEM from three independent experiments. As a reference, the NO ORF %IP values for each strain were as follows: yADP75: 1.60 ± 0.603, yADP76: 2.04 ± 0.066.

To further characterize the effects of the H4-R36A mutant on the transcription elongation process, we carried out additional ChIP experiments to determine the effects of this histone mutant on Pol II and bulk nucleosome occupancy across the *PMA1* and *FBA1* genes. In these experiments, we found that the H4-R36A mutant caused a modest reduction in occupancy of both the Pol II subunit Rpb3 and histone H3 at the 5′ ends of *PMA1* and *FBA1* and had no significant effect on the levels of occupancy of the two proteins at the 3′ ends of the same genes [Figures 6 and 7; note that our results on the effects of H4-R36A on histone H3 occupancy at *PMA1* are very similar to those reported in a recent study (Hainer and Martens 2011)]. Because these effects are rather minor compared with the effects imparted by the H4-R36A on Spt16 distribution across the same genes, we interpret these results as evidence that the effects of H4-R36A on transcription elongation events are at least to some degree specific to Spt16. A similar conclusion was reached for the H3-L61W mutant based on similar studies performed in studies we described previously (Duina *et al.* 2007).

## Discussion

In this work, we have shown that two specific histone H4 mutants—H4-R36A and H4-K31E—cause perturbations in the pattern of association of the transcription elongation factor Spt16 across transcribed genes. In particular, we found that these histone mutants cause a shift in the distribution of Spt16 toward the 3′ regions of the transcribed genes we investigated. These effects are similar to those we previously reported for cells expressing the histone H3 mutant H3-L61W, and a closer inspection of the effects conferred by H4-R36A indicates that this histone mutant and H3-L61W likely affect Spt16 association with transcribed genes through perturbations of a common process. These results, combined with the fact that H4-R36, H4-K31, and H3-L61 are located in close proximity to each other on the nucleosome structure, suggest that these histone residues may define a nucleosomal region required for proper Spt16-chromatin interactions during the process of transcription elongation.

Whereas it is clear that the H4-R36A, H4-K31E, and H3-L61W mutants perturb normal Spt16−chromatin interactions during transcription, the mechanism underlying the shift in Spt16 distribution toward the 3′ ends of transcribed genes still remains to be elucidated. A simple interpretation of our results is that the histone mutants affect two aspects of Spt16−gene interactions: first, the mutants impair proper recruitment of Spt16 at the 5′ ends of transcribed genes, and second, the mutants interfere with the proper disengagement of Spt16 over the 3′ regions of the same genes. This model is consistent with our ChIP data showing lower Spt16 abundance at the 5′ends of transcribed genes and abnormally high levels of Spt16 occupancy at the 3′ ends of the same genes relative to the amount recruited over the corresponding 5′ regions. Whereas it would seem likely that the histone mutants interfere with proper Spt16 dissociation from chromatin at the 3′ ends of transcribed genes in a direct fashion, the lower levels of Spt16 occupancy at the 5′ regions of the same genes may be reflective of either a direct impairment in Spt16 recruitment to genes over these regions or an indirect effect stemming from an overall lower concentration of available Spt16 in the nucleoplasm for gene recruitment due to abnormal retention of Spt16 at the 3′ ends of genes genome-wide. We note that implicit to our model is the assumption that Spt16 can only be recruited to the 5′ end of transcribed genes and that it does not disengage from chromatin until it has traveled across the entire length of these genes. However, it is also possible that distinct Spt16 proteins are recruited to and dissociate from chromatin throughout the length of transcribed genes in a dynamic fashion—in this scenario, our histone mutants would be postulated to affect one or more aspects of these dynamic interactions ultimately resulting in abnormal patterns of Spt16 distribution across transcribed genes as we have described.

How might the H4-R36, H4-K31, and H3-L61 residues normally participate in ensuring proper interactions between Spt16 and chromatin during transcription elongation? It could be envisioned that a region of the nucleosome that includes these residues participates in direct interactions with yFACT and that these interactions are in some way required for proper yFACT disengagement from, and possibly recruitment to, chromatin during the transcription process. The contribution of each of the three histone residues in ensuring proper Spt16−chromatin interactions is yet to be defined. Within the context of an intact nucleosome, the H4-R36 residue directly contacts the DNA (Luger *et al.* 1997; Luger and Richmond 1998) and thus it is possible that this DNA−histone interaction is an important feature of the nucleosome required for efficient interactions between yFACT and chromatin. Alternatively, it is conceivable that H4-R36 makes direct interactions with yFACT at some point during yFACT-mediated manipulations of nucleosomes. The H3-L61 residue is buried and therefore unlikely to make direct contacts with yFACT (at least in the context of an intact nucleosome), but a substitution to a tryptophan could cause a structural perturbation within nucleosomes that would in turn lead to abnormal yFACT-chromatin interactions. It is also possible that the H3-L61W mutant exerts its effects by interfering with the ability of the nearby H4-R36 residue to form proper bonds with the DNA or possibly, as discussed previously, with yFACT itself. Finally, H4-K31 is exposed on the surface of the nucleosome and could possibly interact with yFACT directly.

The H4-R36A mutant was among eight histone mutants identified in a recent study that cause reductions in nucleosome occupancy at several highly transcribed genes (Hainer and Martens 2011). Furthermore, in this and previous studies (Myers *et al.* 2011), we have shown that both H4-R36A and H3-L61W cause cryptic intragenic transcription initiation events at the *FLO8:HIS3* gene [and, in the case of H3-L61W, at the endogenous *FLO8* gene as well (Duina *et al.* 2007)]. Collectively, these observations suggest that these histone mutants might interfere with one or more steps involved in transcription-coupled nucleosome reassembly. Whether this transcription-coupled nucleosome reassembly defect is related to the perturbations in Spt16 association across transcribed genes seen in the context of our histone mutants remains an open question. It could be envisioned, for example, that yFACT-mediated nucleosome reassembly is a required step in ensuring proper disengagement of yFACT from the ends of genes and mutations—in the histones or conceivably in other proteins as well—that interfere with this step would cause accumulation of yFACT at the 3′ ends of genes. Alternatively, it could be proposed that the inability of yFACT to properly disengage from the 3′ ends of genes in the histone mutant backgrounds results in the presence of abnormally elevated levels of stalled yFACT-nucleosome complexes over the 3′ ends of genes, which in turn could be responsible for the cryptic intragenic transcription phenotypes we have observed. In a previous study, however, we obtained results in apparent conflict with these hypotheses as we found that among a battery of newly isolated Spt16 mutants, one of them—Spt16-E735G—was among the strongest suppressors of the H3-L61W−dependent Spt16 3′ accumulation at *PMA1* but was among the weakest suppressor of the cryptic initiation phenotype at *FLO8:HIS3* (Myers *et al.* 2011); thus, at least in this case, dissociation of yFACT from the 3′ end of a gene and nucleosome reassembly appear not to be functionally linked. However, additional genetic and biochemical experiments need to be carried out to more directly assess a possible functional relationship between transcription-coupled nucleosome reassembly and the processes that regulate yFACT disengagement from the ends of genes after transcription.

Collectively, our results show that specific histone mutants can significantly perturb the association patterns of Spt16 across transcribed genes and suggest that a region of the nucleosome that encompasses the H4-R36, H4-K31, and H3-L61 residues on the side of the nucleosome core particle plays an important role in ensuring proper association of yFACT with transcribed genes. Future studies will be directed toward a more complete characterization of this nucleosomal region and will include studies aimed at identifying other residues in the vicinity of the H4-R36, H4-K31, and H3-L61 residues that may contribute to proper yFACT−gene interactions.
